# Application of Low-Cost Electrochemical Sensors to Aqueous Systems to Allow Automated Determination of NH_3_ and H_2_S in Water

**DOI:** 10.3390/s20102814

**Published:** 2020-05-15

**Authors:** Malcolm Cämmerer, Thomas Mayer, Stefanie Penzel, Mathias Rudolph, Helko Borsdorf

**Affiliations:** 1 UFZ—Helmholtz Centre for Environmental Research GmbH, Department Monitoring and Exploration Technologies, Permoserstraße 15, D-04318 Leipzig, Germany; thomas.mayer@ufz.de (T.M.); helko.borsdorf@ufz.de (H.B.); 2 Faculty of Engineering, Leipzig University of Applied Science, Karl-Liebknecht-Str. 134, D-04277 Leipzig, Germany; stefanie.penzel@htwk-leipzig.de (S.P.); mathias.rudolph@htwk-leipzig.de (M.R.)

**Keywords:** chemical sensors, humidity, water quality

## Abstract

Usage of commercially available electrochemical gas sensors is currently limited by both the working range of the sensor with respect to temperature and humidity and the spikes in sensor response caused by sudden changes in temperature or humidity. Using a thermostatically controlled chamber, the sensor response of ammonia and hydrogen sulfide sensors was studied under extreme, rapidly changing levels of humidity with the aim of analyzing nebulized water samples. To protect the sensors from damage, the gas stream was alternated between a saturated gas stream from a Flow Blurring® nebulizer and a dry air stream. When switching between high and low humidity gas streams, the expected current spike was observed and mathematically described. Using this mathematical model, the signal response due to the change in humidity could be subtracted from the measured signal and the sensor response to the target molecule recorded. As the sensor response is determined by the model while the sensor is acclimatizing to the new humid conditions, a result is calculated faster than that by systems that rely on stable humidity. The use of the proposed mathematical model thus widens the scope of electrochemical gas sensors to include saturated gas streams, for example, from nebulized water samples, and gas streams with variable humidity.

## 1. Introduction

Low-cost electrochemical sensors have been widely used to monitor air quality or ensure industrial safety. These sensors can usually only be applied to normal air analysis as they are prone to either drying out when the humidity remains too low or bursting when the humidity remains too high. The signal produced by these sensors is also dependent on a wide range of factors other than the compound under investigation. These include temperature, humidity, supply voltage, and other compounds present in the gas mixture [1]. Despite these problems, the advantages of low cost; continuous; and, where required, high density measurements have led to electrochemical sensors being used in urban and industrial settings [2,3,4].

Two of the compounds being monitored with air quality sensors are ammonia and hydrogen sulfide. These are routinely monitored using a wide variety of sensors owing to their potential to cause unpleasant and harmful odors [5,6,7,8]. However, as ammonia and hydrogen sulfide are both water soluble, forming the ammonium and hydrosulfide ions, the risks posed by these compounds are not limited to the gaseous environment. The odorant emission capacity of water can be characterized (VDI 3885 Blatt 1:2017-06) as well as the concentration of ammonium and sulfides in the water. The standard photometric measurement of ammonium and sulfides (DIN ISO 15923-1 (D49) and DIN 38405-D27 (D27)) are off-line measurements that require specific chemical reagents as well as a photometer for measurements. Although efforts have been made to automate the sampling and measurement process [9], the development of a system based on low-cost electrochemical sensors could achieve the following: drastically reduce costs, eliminate the need for chemical reagents, and increase the frequency of measurement.

To allow the use of low-cost electrochemical sensors for online water analysis, the compounds under investigation must be continuously extracted from the sample liquid. This means that large volumes of a sample will be continuously extracted. Such a system is advantageous when considering environmental probes taken from large, potentially heterogeneous samples as the accuracy of the measurement is greatly influenced by the ratio of probe to sample volume and the number of measurements taken [10,11]. Taking a large sampling volume of water reduces the inaccuracies associated with sampling a large body of water and the effect of heterogeneity. Alternatively, a large number of small samples (with a larger expected standard deviation owing to heterogeneity in the bulk sample) can be considered [11], which then allows for alternative extraction methods to be used.

Traditionally, purge and trap methods have been used to reduce the volume of gas for analysis while retaining the advantage of larger sample volumes (e.g., 200 mL [12]). Alternatively, headspace extraction, potentially coupled with solid phase microextraction (SPME), allows the sample volume to be reduced too (e.g., 3 mL [13]). The advantage of smaller sampling volumes is that the measurement frequency can be increased. The disadvantage of sample concentration with SPME is that the measurement is no longer an online measurement, despite the ease of taking multiple samples.

Depending on the species under investigation, the extraction of compounds into air can be encouraged by adjusting the pH. As hydrogen sulfide and ammonia exist in equilibrium in solution with their respective ionic forms, the proton concentration will have a large effect on the equilibria shown here:

(1) $$HS^{-}+H^{+}↔H_{2}S,$$

(2) $$NH_{4}^{+}↔NH_{3}+H^{+}.$$

By lowering the pH of water containing sulfide ions, hydrogen sulfide will be formed and then released. Conversely, ammonia by will be formed and then released by raising the pH of water containing ammonium ions. These equilibria also have an important role to play when defining the maximum allowable concentration of, for example, sulfide ions in water. In this case, the concentration will normally be measured at pH = 4 (DIN 38405-D27 (D27)).

An alternative method to increase the concentration of volatile compounds in the headspace above a sample is to reduce the pressure. Again, the equilibrium of the sample will be disturbed and Henry’s Law shows that the concentration of a substance dissolved in a liquid is proportional to the partial pressure of the gaseous substance above the liquid [14,15]. Therefore, as the partial pressure of a substance above a liquid decreases, the concentration of that substance dissolved in the liquid decreases and it will be extracted into the low pressure gas. This has been used to allow the real-time monitoring of ammonia in water using infrared spectrometry [16]. As Henry constants are temperature-dependent, changes in the temperature can also be used to increase the vapor pressure of the headspace gas and affect its composition.

The equilibrium between the gas above a liquid and the liquid itself can also be disturbed by constantly replacing the gas above the liquid with a gas containing a lower concentration of the substance to be extracted (ideally a pure gas). In this case, the partial pressure of the dissolved substance is reduced so that the dissolved substance is released from the liquid in an attempt to restore the equilibrium. This effect is the basis of the stripping that is recommended in VDI 3885. Mass transfer rates between liquid and gas phases are proportional to the difference in mole fraction of a substance per unit area [17]. Therefore, a large surface area created by the multitude of small gas bubbles within the liquid promotes transfer of the dissolved compound to the gas.

The surface area between the liquid gas phases can equally be increased by dispersing liquid droplets in a bulk gas. This can be achieved using a nebulizer and, depending on the type of nebulizer used, there can be large differences in the maximum possible surface area. The advantage of using a nebulizer compared with stripping a large volume of water with a large volume of gas is that it could allow continuous analysis. The footprint of a nebulizer and pumps is also smaller than that of a 30 L container, meaning that a nebulizer-based setup may be more compact and easier to transport. Gano-Calvo showed that Flow-Blurring® nebulizers offer more efficient atomization in comparison with conventional pneumatic nebulizers [18,19]. As atomization efficiency is inversely proportional to droplet diameter, this will lead to a large surface area of contact between the liquid and gas phases. When combined with appropriate temperature, pressure, and pH control, a nebulizer-based extraction of dissolved substances into the gas phase could allow continuous monitoring of water samples using low-cost electrochemical sensors.

While commercially available ion selective electrodes designed for use in water offer a simpler alternative to determine the levels of ammonium [20] and sulfide [21,22], these electrodes typically cost between 500 and 1000 €. Such electrodes have been used to create, for example, depth profiles in lakes [23,24,25]. Low-cost electrochemical gas sensors are, however, available for under 100 € and do not require a separate reference electrode as the reference electrode is housed within the sensor. While efforts have been made to reduce the cost of aqueous electrochemical measurements [26], such sensors are not commercially available. The use of low-cost electrochemical gas sensors is thus more cost effective, if they can be used effectively in a high humidity environment.

Another alternative technique, which has been successfully applied to both high humidity gases and aqueous systems, is to measure the change of mass using a Quartz crystal microbalance (QCM). By coating the surface of the QCM, specific sensors for ammonia/ammonium ions [27,28] or hydrogen sulfide [29,30] have been developed. While QCMs are easily available, the measurement of specific species is only possible if the users are able to apply the specific coating to the surface of the QCM themselves. This currently presents a barrier to the widespread use of these specific, easily miniaturized sensors.

Therefore, to investigate the possibility of using low-cost electrochemical gas sensors to specifically measure the levels of ammonia and hydrogen sulfide in water samples, a metallic nebulizer was constructed to allow the continuous extraction of water samples at a defined temperature. The extracted gas stream was then analyzed using an algorithm to decouple the effect of humidity from the effect of target molecules on the low-cost electrochemical sensors. This was integrated into a custom built user interface to allow online analysis of water samples.

## 2. Materials and Methods

Two electrochemical sensors were selected for their appropriate working range and sensitivity: H2S A4 Hydrogen Sulfide 4-Electrode Sensor (for concentrations under 50 ppm, 0.171 nA/ppb) and NH3 AF Ammonia 3-Electrode Sensor (for concentrations under 100 ppm, 0.360 nA/ppb) (Alphasense Ltd., Great Notley, UK). These were mounted on an 810-0021-03 2-Way Analog Front End (Alphasense Ltd., Great Notley, UK), which allowed the power source and signal connections to be easily achieved. The analog signal was processed by an ADS1115IDGST (Texas Instruments, Dallas, TX, USA). This digital signal was then transferred to a laptop via an Arduino UNO SMD Edition (Arduino SA, Chiasso, Switzerland). The pH of buffer solutions was determined using a Mettler Toledo Five easy (Mettler-Toledo GmbH, Gießen, Germany).

Analytical grade ammonia 25%, sodium bicarbonate, sodium hydroxide, and sodium iodide (Merck KGoA, Darmstadt, Germany) were used to mix solutions with water from a Milli-Q^®^ Direct 8 (Merck KGoA, Darmstadt, Germany). Additionally, sodium chloride of analytical grade (Fisher Scientific GmbH, Schwertewas, Germany) was used. Sodium sulfide 35% and hydrochloric acid (Merck KGoA, Darmstadt, Germany) were mixed dropwise to create hydrogen sulfide, which was bubbled through water from a Milli-Q^®^ Direct 8 to provide hydrogen sulfide solutions.

### 2.1. Sensor Chamber

**Figure 1:**
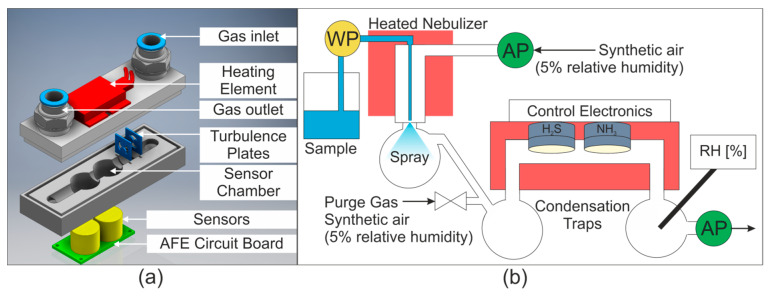
(**a**) Sensor chamber. AFE = Analog Front End. (**b**) Gas and water flow in system. WP = water pump, AP = air pump, RH = relative humidity sensor. Temperature controlled areas are shaded in red. The purge gas valve was opened to dry the sensors between measurements.

To minimize condensation within the sensor chamber while using warm and humid air streams, a heated sensor chamber was constructed from stainless steel. The temperature range of the sensors is −30 to 50 °C, so a PTC (Positive Temperature Coefficient) Heating Element FG14756.1 (DBK David + Baader GmbH, Kandel, Germany) was selected as it delivers a surface temperature of 40 °C. This was screwed onto the surface of the stainless steel sensor chamber and delivered a profile temperature of approximately 33 °C. The sensor chamber is shown in Figure 1a.

### 2.2. Nebulizer and Gas Transport

As previously mentioned, Flow Blurring^®^ nebulizers are affordable high efficiency nebulizers. Two Flow Blurring^®^ nebulizers were used in this study. Firstly, a OneNeb Inert Concentric Nebulizer (Aglient, Santa Clara, CA, USA) was used for the initial studies. Later, a stainless steel nebulizer with similar characteristics (0.5 mm inner diameter of liquid tube) was constructed based on the information provided by Gano-Calvo [18]. This nebulizer could be heated using flexible polyimide coated heaters to allow the effect of temperature on the extraction process to be investigated.

Water was delivered to the nebulizer using a WellChrom HPLC-Pump K-1001 (KNAUER Wissenschaftliche Geräte GmbH, Berlin, Germany). The gas stream was pumped into the nebulizer with a 1410VD/1.5/E/BLDC Pump (Gardner Denver Thomas GmbH, Fürstenfeldbruck, Germany). The airflow was then drawn through the sensor chamber by a 3003VD/0,7/E/DC Pump (Gardner Denver Thomas GmbH, Fürstenfeldbruck, Germany). The gas and water flows are shown schematically in Figure 1b. The relative humidity of the gas stream was measured downstream from the sensor chamber using a Testo 435 equipped with a humidity probe (Testo SE & Co. KGaA, Titisee-Neustadt, Germany).

### 2.3. Data Acquisition and Processing

A data acquisition program was written using Python™ to display, manage, and analyze the serial data sent to the laptop from the ARDUINO^®^ board. The standard data acquisition procedure was to measure data points at an approximate frequency of 10 Hz and display the average of two data points. This meant that five new data points were created approximately every second. The mathematical model used to separate the signal owing to ammonia or hydrogen sulfide from the signal caused by changes in humidity was integrated into the user interface so that these parameters could be monitored online.

## 3. Results

Before the effectiveness of low-cost electrochemical sensors in an extremely humid gas stream could be assessed, it was first necessary to understand the influence of this humid environment on the sensors. The sensors have a stated working range of 5–95% relative humidity, and working outside of this range can have severe consequences. For example, prolonged exposure to high humidity can cause the electrolyte to burst the sensor, which will then need to be replaced. As the relative humidity of the gas stream from the nebulizer was over 95%, the sensors were first dried with synthetic air (relative humidity < 5%) before being exposed to the gas stream from the nebulizer. These drastic changes to the humidity of the gas stream over the sensors lead to significant changes in the baseline signal, which first needed to be accounted for before any analysis could be performed on the signal.

### 3.1. Sensor Response and Effect of Humidity

**Figure 2:**
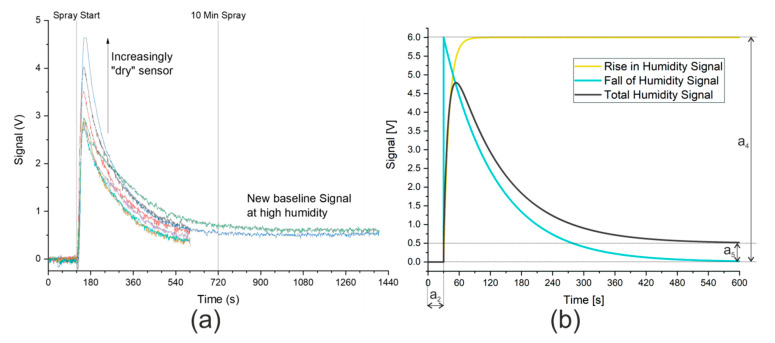
(**a**) Signal from ammonia sensor owing to gas stream saturated with water. Synthetic air with 5% relative humidity (purge gas) measured until spray start at 120 s. Saturated gas stream then continuously measured until end of data. Signal from hydrogen sulfide sensor is analogous and is shown in Figure S5. Ten min spray time marked to show the manufacturer’s recommended time to acclimatize to new humidity level. (**b**) Model describing signal caused by humidity. Coefficients: a_2_ = 30 s, a_4_ = 6 V, a_5_ = 0.5 V. Rise in humidity signal modeled against humidity measurements, as shown in Figure S6.

Alphasense notes in AAN 110 [31] that current spikes are caused by even small changes in humidity. A sudden change to a higher humidity causes a positive spike that incorrectly suggests that more of the substance under investigation is present. A spike in the other direction, suggesting that the concentration has decreased, is observed when the humidity is suddenly lowered. Although the sensors will acclimatize to the new humidity level, this can take over 10 min, as shown in Figure 2a. A small offset in the signal was nevertheless observed at this extreme level of humidity. Unfortunately, the larger the change in humidity, the larger the spike caused by the change. This accounts for the differing heights of the signal spike seen in Figure 2a. After repeated cycles of humid and dry gas streams of constant duration, the sensor reached a quasi-steady state, where a constant spike owing to an increase in humidity was observed (approximately 3 V in Figure 2a). The signal from the hydrogen sulfide sensor in the presence of a saturated gas stream is analogous and shown in Figure S5.

A simple method to measure the signal from ammonia or hydrogen sulfide would be to expose the sensor to the humid air containing the sample and wait a sufficiently long time (more than 10 min) before taking the measurement. This approach, however, puts the sensors at risk of bursting as the sensors are exposed to gas saturated with water for over 10 min. All the data points captured while the sensors acclimatize to the new conditions are also discarded. The use of a mathematical model to describe the signal change owing to the change in humidity would allow all data points to be considered and potentially increase the accuracy of the measurement. In addition, if the mathematical model could describe the effect of humidity sufficiently well, the measurement time could be reduced as the real signal could be analyzed while the sensor was acclimatizing. This would both protect the sensor from bursting and allow more measurements to be taken.

### 3.2. Development and Validation of Mathematical Model

A mathematical model was thus developed that described the effect of humidity on the signal and allowed the direct analysis of the ammonia or hydrogen sulfide signal. The graphical description of the signal caused by humidity is shown in Figure 2b. Both the data provided by Alphasense and the initial measurements using the nebulizer show that, in the presence of humid air, the signal rises rapidly to a maximum before returning exponentially to a minimum. The maximum was described in the mathematical model by a parameter, a_4_, which would need to be fitted to allow for different sensor “dryness”. The offset in the signal owing to humidity is also fitted using a parameter, a_5_. Finally, the time when the spray started was set to zero in the model using a further parameter, a_2_. The mathematical descriptions of the curves displayed in Figure 2b are shown here:

(3) $$Modelled\;Signal\;\left(Total\;humidity\;signal\right)=e^{\left(-a_{3}\left(t-a_{2}\right)\right)}\cdot a_{4}\left(1-e^{\left(-a_{6}\cdot \left(t-a_{2}\right)\right)}\right)+a_{5},$$

(4) $$Modelled\;Signal\;\left(Fall\;of\;humidity\;signal\right)=e^{\left(-a_{3}\left(t-a_{2}\right)\right)}\cdot a_{4},$$

(5) $$Modelled\;Signal\;\left(Rise\;in\;humidity\;signal\right)=a_{4}\left(1-e^{\left(-a_{6}\cdot \left(t-a_{2}\right)\right)}\right),$$

(6) $$Modelled\;Signal\;\left(Offset\;humidity\;signal\right)=a_{5}.$$

The values a_2_, a_4_, and a_5_ are shown graphically in Figure 2b and their effects and values can be easily inferred. The offset in time represented by a_2_ is 30 s and, by subtracting this value from the time, the model has a start point where the *e*-function is raised to the power zero, and thus equals 1. This means that, according to Equation (4), the falling signal starts at a_4_ before dropping to 0 V. In Equation (5), the rising signal starts at 0 V and increases to a_4_. In the example in Figure 2b, a_4_ has a value of 6 V. As time increases, the *e*-function tends toward zero with the rate of change determined by two additional coefficients, a_3_ and a_6_. The smaller these coefficients, the slower the change. Here, a_3_ is ten times smaller than a_6_, meaning that the *e*-function of the falling edge requires significantly longer to reach zero than the *e*-function of the rising edge. The final coefficient shown in Figure 2b is a_5_, the offset in the signal at high humidity, which is shown taking a value of 0.5 V. A final consideration of the model is that when (t − a_2_) < 0, that is to say, before the model’s start time, the model takes the default value of zero.

**Figure 3:**
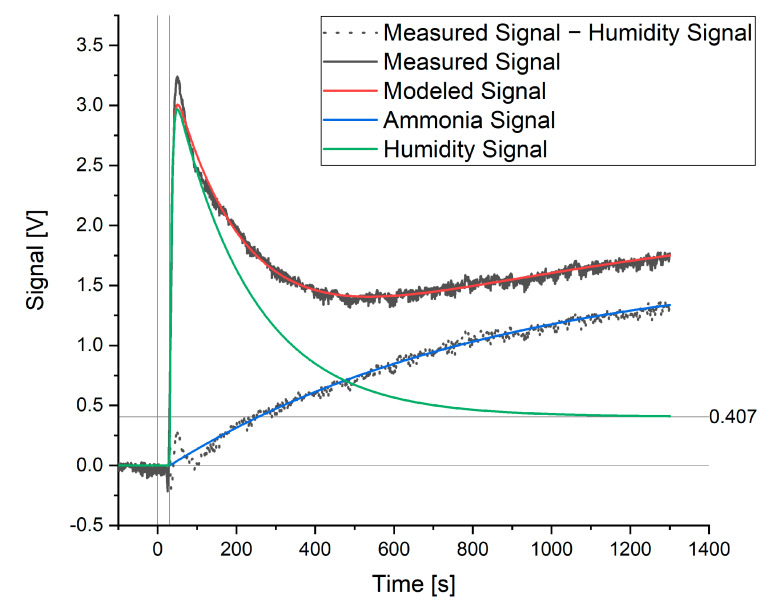
Modeled and measured signals for a sample containing 60 ppm ammonia.

When modeling a signal where ammonia or hydrogen sulfide was present, a further element of the signal was detected. This additional signal is shown in Figure 3 as the measured signal minus the humidity signal. As a known amount of ammonia had been added to the water sample and this extra signal was not shown by the hydrogen sulfide sensor, this signal was attributed to ammonia.

This signal can be modeled in the same way as the rise in the humidity signal (Equation (5)). When the signal maximum and rate constant for the ammonia signal (a_0_ and a_1_) were fitted to the data, the measured and modeled signal showed good agreement, as seen in Figure 3. The rate constant for the rise in the ammonia signal was then fixed (independent of ammonia concentration) and the maximum, a_0_, was taken as the theoretical maximum signal for ammonia that would be achieved at equilibrium. The value of this a_0_ coefficient was thus used when creating calibration curves with respect to ammonia or hydrogen sulfide and for the further optimization of this method. The complete model is as follows:

(7) $$Modelled\;Signal=a_{0}\left(1-e^{\left(-a_{1}\cdot \left(t-a_{2}\right)\right)}\right)+e^{\left(-a_{3}\left(t-a_{2}\right)\right)}\cdot a_{4}\left(1-e^{\left(-a_{6}\cdot \left(t-a_{2}\right)\right)}\right)+a_{5}.$$

t: Time
a_0_: Theoretical maximum signal due to ammonia or hydrogen sulfide
a_1_: Rate constant of rise in signal due to ammonia or hydrogen sulfide
a_2_: Start point of the data (time = 0)
a_3_: Rate constant of drop in signal as sensor acclimatizes to new humidity
a_4_: Maximum signal caused by humidity
a_5_: offset in signal due to humidity
a_6_: Rate constant of rise in signal as sensor acclimatizes to new humidity

The speed of calculation and reliability of the model can be increased by considering the logical boundaries for the coefficients a_2_–a_6_. All coefficients are limited to positive values and the following boundaries were set after observing the signal response to the humid gas stream from the nebulizer. The offset in time depends on how the data are presented to the model. As a reference time of 10 s before the rising edge (t_0_) is used, a_2_ may vary between 2 and 20 s. The data are also compared to the average signal in the 50 s before t_0_, so that the signal is approximately zero before the rising edge. As the maximum offset caused by humid air was approximately 0.3 V, a_5_ may vary between 0 and 0.4 V. Finally, as the minimum signal caused by humid air is greater than 2 V and the signal from the ADS1115IDGST is saturated at 5 V, a_4_ may be modeled between 2 and 8 V. As a signal caused by ammonia or hydrogen sulfide will only be detected if the humidity signal falls away (both in the model and in reality), the rate constant a_3_ observed when measuring humid gas is used as a guide for the lower boundary. In the case of the ammonia sensor, this is 0.0035. This defines the slowest rate that the curve can fall at, and thus any signal over this modeled curve is attributable to the compounds of interest.

The model coefficients are optimized using the curve_fit function from the SciPy package in Python™. The “Trust Region Reflective” least squares method was used to find the local minimum of the function within the boundaries discussed previously. When the a_1_ coefficient is large, the offset due to humidity, a_5_, and the signal caused by the substance of interest, a_0_, have a similar effect on the model. As a result, coefficient a_1_ was optimized for ammonia and hydrogen sulfide and held constant for the following measurements. This choice also reduced the degrees of freedom in the model and allowed faster optimization of the other parameters. The new equation for modeling the ammonia signal becomes the following:

(8) $$Modelled\;Signal=a_{0}\left(1-e^{\left(-0.001204\cdot \left(t-a_{2}\right)\right)}\right)+e^{\left(-a_{3}\left(t-a_{2}\right)\right)}\cdot a_{4}\left(1-e^{\left(-a_{6}\cdot \left(t-a_{2}\right)\right)}\right)+a_{5}$$

The effectiveness of the model at isolating the influence of ammonia or hydrogen sulfide on their respective sensor signals can be evaluated by looking at the relationship between the a_0_ coefficient and the concentration of ammonia or hydrogen sulfide in the sprayed solution. The linear relationship (R^2^ > 97% for both ammonia and hydrogen sulfide) that was observed and shown in Figures S2 and S3 shows that the model works well. The calibration curve for ammonia showed that the limit of detection was approximately 10 ppm, which is sufficiently low for the detection of ammonia in environmental water samples.

Despite the boundaries are put on the model, the model still has a large number of parameters that can be changed to describe measured signal. The model thus requires eight minutes of data to show a reproducible and highly linear relationship between a_0_ and concentration for the substance under investigation. The relationship between the number of data points used for the measurement and the coefficient of determination is shown in the Supplementary Materials. Shorter measurement times are possible, however, the model does not have enough data to reliably estimate a_0_ or rather the concentration of ammonia or hydrogen sulfide. Nevertheless, the use of this mathematical model provides a 20% saving in the measurement time compared with the 10 min suggested by Alphasense after a change in humidity level. When using the sensors under normal conditions, Alphasense states that 90% of the signal maximum is reached in less than 60 s. This, however, means that measurements at equilibrium would take more than twice as long (more than 2 min). Considering that the signal caused by ammonia was still rising after 20 min and that this continuous increase in the signal was only seen in the presence of ammonia or hydrogen sulfide, the interaction of ammonia and hydrogen sulfide with the sensor appears to have changed. This could be because of the formation of a film of water on the membrane of the sensor, which slows the process of reaching equilibrium. Nevertheless, under these extreme conditions, a reduction of the measurement time to eight minutes offers a huge time saving compared with a measurement at equilibrium. It is also possible to consider all the data points in the data set, rather than just looking at the data points after 10 min, when the effect of humidity is assumed to be minimal, or greater than 20 min, when the signal reaches a maximum.

A further benefit of this model is that it could be integrated into the Python™ user interface, used to collect the data from the sensor control board. As a result, the model could be optimized after each new data point and the optimized a_0_ coefficients (representing the signal intensity at equilibrium) for ammonia and hydrogen sulfide displayed in the console output. This model thus shows great potential for integration into automated measurement systems.

### 3.3. Optimization of Measurement Conditions (Water Flow, Temperature, Ionic Strength, and pH)

**Figure 4:**
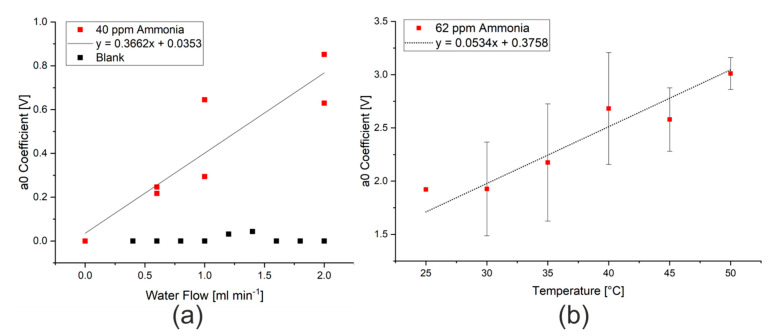
(**a**) Effect of water flow rate on the a_0_ coefficient measured when spraying 40 ppm ammonia in water. (**b**) Effect of nebulizer temperature on the a_0_ coefficient measured when spraying 62 ppm ammonia in water at 2 mL min^−1^.

As previously discussed in the introduction, there are a range of parameters that could affect the efficiency of extraction, and thus the limit of detection of the procedure. Changing the rate of water flow to the Flow Blurring^®^ nebulizer changes the air to water ratio, which will affect both the size of droplets produced by the nebulizer as well as the concentration gradient between the water droplets and the air during extraction. By increasing the water flow rate, larger droplets with a smaller surface area to volume ratio will be produced and the extraction into the gas phase will be less successful. The total amount of ammonia or hydrogen sulfide that can be potentially extracted will, however, increase, as would be expected when changing the phase ratio in a headspace extraction. After the signal was modeled and the effect of humidity eliminated, this increase in the available substance for extraction appears to be the major effect, as can be seen in Figure 4a. The use of the mathematical model thus allows the extraction to take place at the manufacturer’s maximum recommended flow rate (2 mL min^−1^) without risk of damaging the sensors. A water flow rate of 2 mL min^−1^ was thus used for the subsequent tests.

The influence on extraction temperature could also be studied using the stainless steel nebulizer, which was constructed in-house. Here, the water flowing into the nebulizer and the body of the nebulizer itself were heated using two small polyimide heating sheets with thermostatic control, which were both set to the same temperature. Changes in the temperature resulted in changes in the modeled ammonia signal intensity (a_0_ coefficient), as shown in Figure 4b. This change in the intensity of the ammonia signal is consistent with the temperature dependence of Henry’s law, where the solubility of dissolved gases decreases with temperature [15,32]. The temperature range that was investigated was, however, too narrow to clearly see the expected relationship that the logarithm of the partial pressure (represented by the measured signal) is proportional to the inverse of the temperature. A temperature of 50 °C was used for further measurements as the highest signal intensities were measured at this temperature and the heating elements did not have sufficient power to achieve higher temperatures.

**Figure 5:**
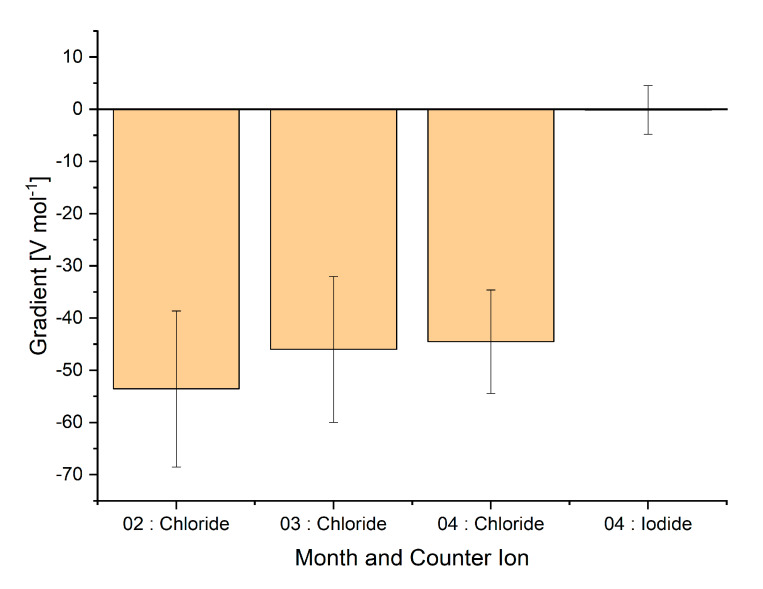
Gradient of the linear regression (y = signal (V), x = salt concentration (mol)). Four different series of measurements are shown, each containing at least nine measurements. Measurements were taken in February, March, and April 2020.

The effect of ionic strength on the signal intensity was also investigated by varying the concentration of sodium chloride or sodium iodide in the water between 0 and 20 mM. This was up to six times the concentration of ammonia in the water sample. Owing to aging of the sensors and pumps controlling the air flow, a decrease in maximum signal intensity was observed with time and between cleaning intervals for the pumps (see Figure S4). Nevertheless, the decrease in signal intensity when the sodium chloride concentration was increased was relatively consistent over a period of three months, as shown in Figure 5. When these measurements were repeated with sodium iodide as the salt, no statistically significant decrease in signal was observed. This suggests that salting in could be taking place with respect to ammonia in the presence of chloride ions [33]. This is possibly because of the formation of ammonium chloride and sodium hydroxide complexes, whereby the equilibrium between ammonia and ammonium ions is disturbed. Clearly, the chemistry of the water in a real sample has a significant effect on the signal that is measured at a certain concentration of ammonia. It would thus be necessary to calibrate the signal response with standard addition of ammonia when working with real samples.

The influence of the matrix on the measurement also creates difficulties when using buffer solutions to control the pH of the water under investigation. In a carbonate/bicarbonate buffer system, changes in the composition of the buffer lead not only to changes in the pH, but also to the concentrations of carbonate and bicarbonate ions in the solution. Measurements were thus conducted varying the concentration and pH of a sodium bicarbonate solution. As the concentration of sodium bicarbonate was increased, the a_0_ coefficient measured reduced. As the concentration of sodium bicarbonate increases, the equilibrium shown in Equation (9) will be shifted to the right and more ammonium ions will be formed:

(9) $$HCO_{3}^{-}+NH_{3}\;↔\;CO_{3}^{2-}+NH_{4}^{+}.$$

**Figure 6:**
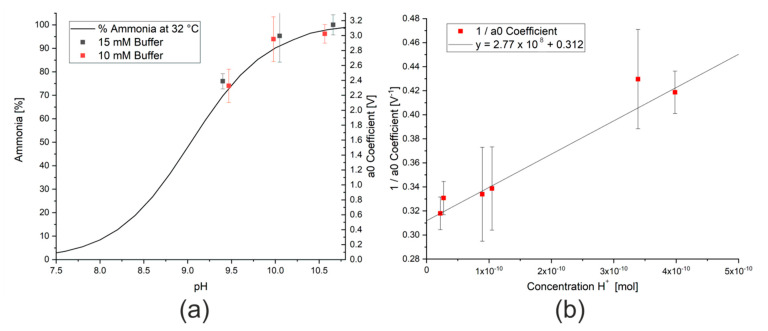
(**a**) Effect of changing pH on percentage of ammonia present in a solution of ammonia molecules and ammonium ions from literature data [36] and data from this study. (**b**) Inverse of a_0_ coefficient measured for a 62 ppm ammonia solution in the presence of different buffer solutions.

This decrease in signal can, however, be compensated by adding sodium hydroxide to the bicarbonate solution to form a carbonate/bicarbonate buffer system. When sufficient buffer (over 10 mM) was present, the results were independent of concentration. As previously discussed, more un-ionised ammonia is formed at higher pH values (Equation (2)), which will lead to a higher signal from the ammonia sensor and a larger modeled a_0_ coefficient. As the pH range for a carbonate/bicarbonate buffer system is approximately between 9 and 11, depending on the temperature and composition [34], the expected pH range is on the right hand side of Figure 6a. The data points from the measurements with carbonate/bicarbonate buffer solution have a similar shape to the literature data. By reforming the equation for the acid dissociation constant, K_a_, as shown in Equations (10)–(14) and taking the a_0_ coefficient as a proxy for the concentration of ammonia, these data points display a linear relationship, as shown in Figure 6b. Analysis of the slope and intercept of the linear regression allows both the maximum a_0_ coefficient for this concentration of ammonia, a_0max_, and K_a_ for ammonia at this temperature to be calculated. In this case, the maximum a_0_ coefficient for 62 ppm ammonia is 3.3 V and the acid dissociation constant at 50 °C is approximately 8. Although this is only an estimation, this value correlates with those from the literature, which show a reduction in K_a_ with increasing temperature [35].

(10) $$K_{a}=\frac{\left[NH_{3}]\cdot [H^{+}\right]}{\left[NH_{4}^{+}\right]}$$

(11) $$[NH_{3}]=\frac{\left[NH_{4}^{+}\right]\cdot K_{a}}{\left[H^{+}\right]};\;\;where\;\;\left[NH_{4}^{+}\right]=\;{\left[NH_{3}\right]}_{max}-[NH_{3}]$$

(12) $$\frac{\left[NH_{3}\right]}{{\left[NH_{3}\right]}_{max}}=\frac{K_{a}}{\left[H^{+}\right]+K_{a}}$$

(13) $$\frac{1}{\left[NH_{3}\right]}=\frac{\left[H^{+}\right]}{{\left[NH_{3}\right]}_{max}\cdot \;K_{a}}+\frac{1}{{\left[NH_{3}\right]}_{max}};\;\;where\;\;[NH_{3}]=\;a_{0}\;\;\;and\;\;\;{\left[NH_{3}\right]}_{max}=a_{0_{max}}$$

(14) $$\frac{1}{a_{0}}=\frac{\left[H^{+}\right]}{a_{0_{max}}\cdot \;K_{a}}+\frac{1}{a_{0_{max}}}$$

### 3.4. Potential for Automation

**Figure 7:**
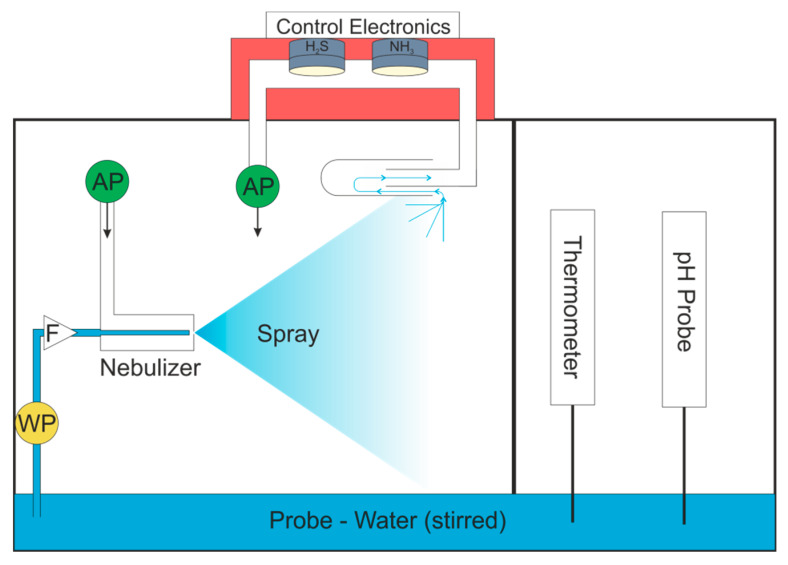
Automated system for real world water quality testing. WP = water pump; AP = air pump; F = filter.

As the Python™ user interface can run the model live, there is no need for offline data processing and the system could potentially be automated and the coefficients of the model saved, used for process control, or sent to cloud based storage. The pump control could be integrated into the Python User Interface so that the start and end times of measurements are correctly identified. This system could use the normal atmosphere to feed air to the nebulizer, if the humidity was first reduced. This could be achieved with a cold trap held at a constant temperature. For monitoring of a natural body of water, the system could either draw the probe into the closed box, as shown in Figure 7, or potentially be floated on the surface of the body of water.

Alternatively, the model presented here could be applied to air quality monitoring in environments where humidity changes frequently, necessitating correction factors for the humidity in the signal. The measurement could be performed using a dried air flow and switched between the dried and humid air flows to allow an internal calibration and subtraction of the signal due to humidity.

## 4. Discussion/Conclusions

This novel application of a mathematical model to allow commercially available air sensors to be used in the aqueous environment seeks to address one of the major issues with these sensors, namely the signal response due to humidity. The use of a mathematical model ensures that the maximum number of data points can be used for the determination of the concentration of an analyte, here, ammonia or hydrogen sulfide, in water while limiting the exposure of the sensors to a potentially damaging level of humidity. The signal measured needs no further calibration for changing humidity and thus eliminates the need for a humidity sensor in the measurement system.

The calculated a_0_ coefficient varies in the expected manner with respect to changes shown in Table 1. The coefficients were optimized in the mathematical model without any changes to the boundaries or the equation, showing that the model is robust and operates in a variety of circumstances.

As a matter of fact, the mathematical model was far more robust than many of the mechanical components in the system (pumps, heaters, nebulizer, and the sensors). The limitations of these components determined the working range of the system and not the mathematical model.

Although the limits of detection achieved in this configuration are not as low as other low cost printed sensors (limit of detection (LOD) 0.34 ppm [37] and LOD 0.43 ppm [26]), these parts cannot simply be ordered from a manufacturer and replaced. The mathematical model developed in this work was also applied in an extreme example and could also be applied to situations within the manufacturer’s specifications, where changes in humidity need to be accounted for. The proposed mathematical model does not, however, remove the need to calibrate for changes in temperature nor does it correct the sensitivity loss that is to be expected with this type of low-cost sensor. A temperature controlled sensor chamber was used to minimize the effect of temperature changes. Owing to the loss in sensitivity quoted by the manufacturer (<3% loss in sensitivity per year for ammonia sensor, <20% loss in sensitivity per year for hydrogen sulfide sensor), data were not directly compared with other measurements acquired more than one month before or after.

It should also be noted that, owing to the respective acid dissociation constants of ammonia and hydrogen sulfide, only one of these compounds can be measured under ideal conditions. The sensors also showed no detectable cross sensitivity under these extreme conditions. The linear relationships observed between the modeled a_0_ coefficient and the concentration of ammonia or hydrogen sulfide (see Figures S2 and S3) show that the mathematical model presented here is applicable to both reducing and oxidizing gas sensors.

While the total cost of the system including all pumps, sensors, and signal transmission to a computer is approximately 1500 €, the greatest costs are those of the nebulizer and pumps, which account for approximately 70% of the total cost. This system is thus significantly less expensive than the photometric systems normally used in the analysis of water samples. Although the data used to assess the effectiveness of the mathematical model presented here were acquired from ammonia and hydrogen sulfide sensors, the sensors are low-cost, easy to replace and can be used in arrays with multiple different sensors. As a result, this system could easily be used with any sensors that show a similar response to humidity.

## Figures and Tables

**Table 1:** Relationship between variables and both the expected theoretical signal and the modeled a_0_ coefficient.

Variable.	Expected Change Signal When Variable is Increased	Change in Modeled a_0_ Coefficient
Concentration	Linear increase in signal	Linear increase
Water Flow	Linear increase in signal	Linear increase
Temperature	Linear decrease in ln(signal) with inverse of temperature	Linear increase in signal with temperature ^1^
pH	Linear increase in inverse of signal with proton concentration	Linear increase in inverse of signal with proton concentration

^1^ Temperature range studied too narrow to confirm linear decrease in ln(signal) with inverse of temperature.

## Supplementary Materials

The following are available online at https://www.mdpi.com/1424-8220/20/10/2814/s1, **Figure S1:** The coefficient of determination, R^2^, for the linear regression of the data from standard solutions with respect to number of data points used by the model; **Figure S2:** The individual model curves (red) for the different concentrations of ammonia calculated using 480 data points; **Figure S3:** Calibration curve for H_2_S based on a serial dilution of a stock solution through which a small volume of gaseous H_2_S had been bubbled; **Figure S4:** Influence of sodium chloride and iodide salt concentration on a0 coefficients of 62 ppm ammonia; **Figure S5**: Signal from hydrogen sulfide sensor due to gas stream saturated with water. Synthetic air with 5% relative humidity measured until spray start at 30 s. Signal drop below 0.5 V shows switch to purge gas stream at 5% relative humidity; **Figure S6**: Change in humidity during spray measured by Testo 435 humidity probe. Exponential model rh = 91.73 × (1 − e^(−0.0488 × (t−18))) in the same form as Equation (5).
