# Supplementary material for: Application of Low-Cost Electrochemical Sensors to Aqueous Systems to Allow Automated Determination of NH3 and H2S in Water
Source: Sensors (Basel). 2020 May 15;20(10):2814. doi: 10.3390/s20102814 (PMC7284547; doi:10.3390/s20102814)
Supplement: Supplementary file 1 [file sensors-20-02814-s001.pdf]

sensors

Article

Application of Low-Cost Electrochemical Sensors to Aqueous Systems to Allow Automated Determination of NH_3 and H_2S in Water

Malcolm Cämmerer [1,*], Thomas Mayer [1], Stefanie Penzel [2], Mathias Rudolph [2], and Helko Borsdorf [1]

[1] UFZ - Helmholtz Centre for Environmental Research GmbH, Department Monitoring and Exploration Technologies, Permoserstraße 15, D-04318 Leipzig, Germany; thomas.mayer@ufz.de (T.M.); helko.borsdorf@ufz.de (H.B.)

[2] Leipzig University of Applied Science, Faculty of Engineering, Karl-Liebknecht-Str. 134, D-04277 Leipzig, Germany; stefanie.penzel@htwk-leipzig.de (S.P.); mathias.rudolph@htwk-leipzig.de (M.R.)

* Correspondence: malcolm.caemmerer@ufz.de

Received: 21 April 2020; Accepted: 12 May 2020; Published: date

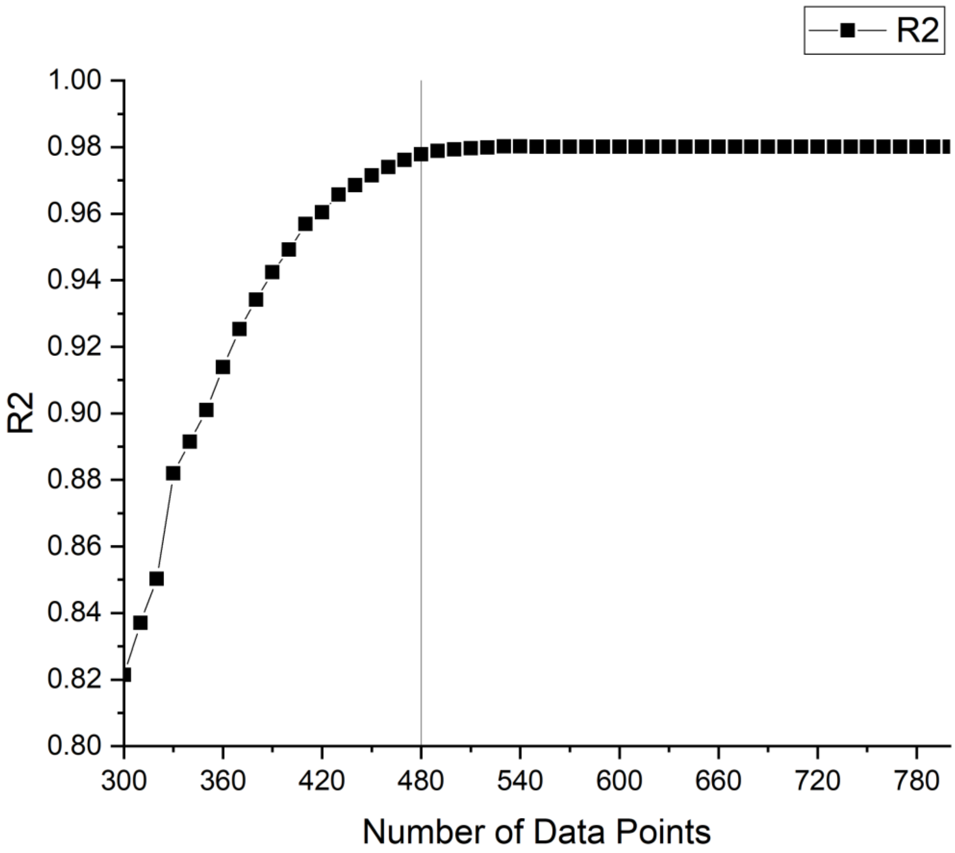

Figure S1. The coefficient of determination, R^2, for the linear regression of the data from standard solutions with respect to number of data points used by the model.

This was recorded for the linear regression of the 23 signals measured with concentrations between 0 and 140 ppm ammonia. The linear regression fits best when the measurement is greater than eight minutes long (480 data points, 1 data point every second).

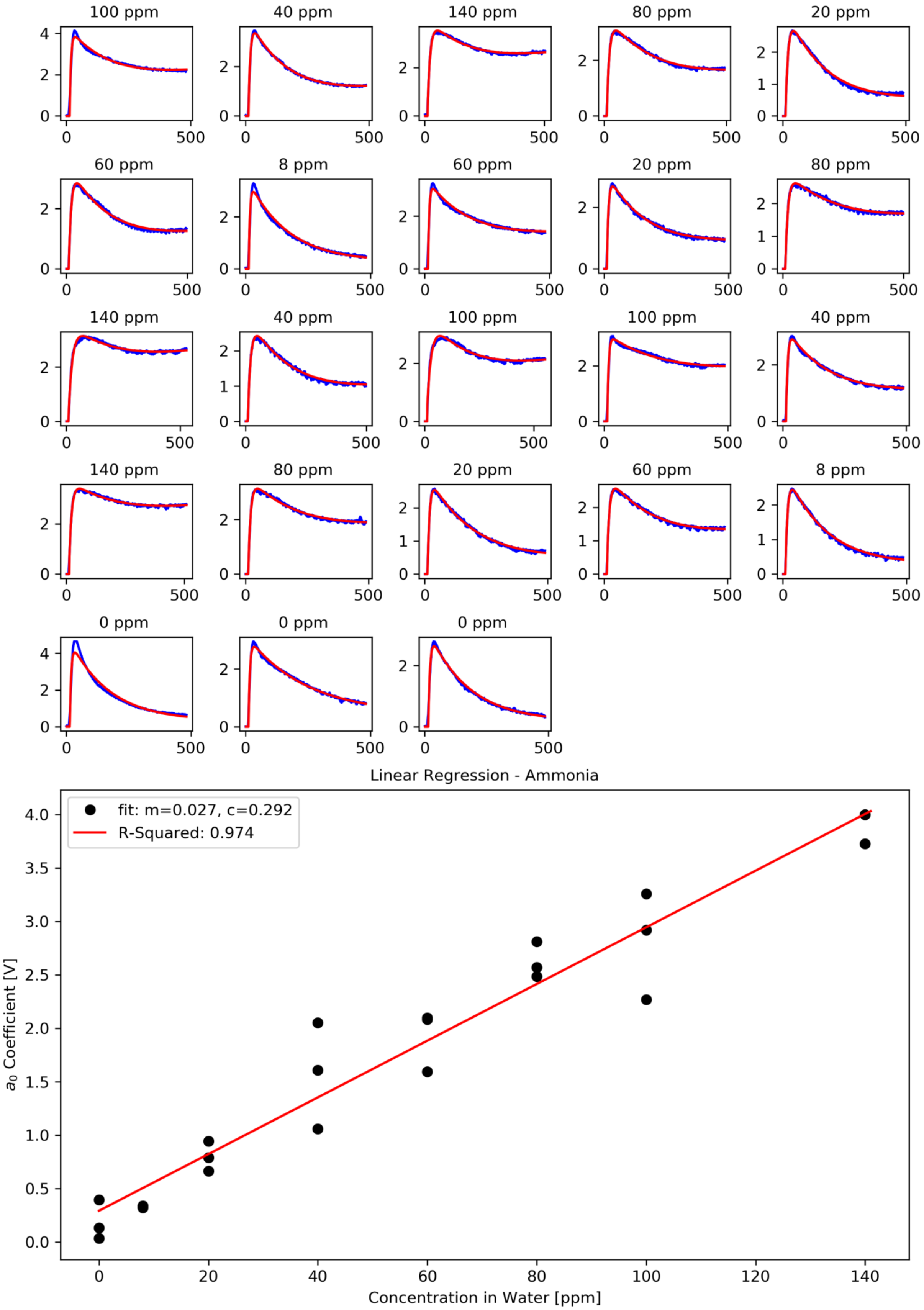

Figure S2. The individual model curves (red) for a 480 s spray of different concentrations of ammonia calculated using 480 data points.

The a_0 coefficients from these modeled curves are shown in the calibration curve at the bottom of the figure.

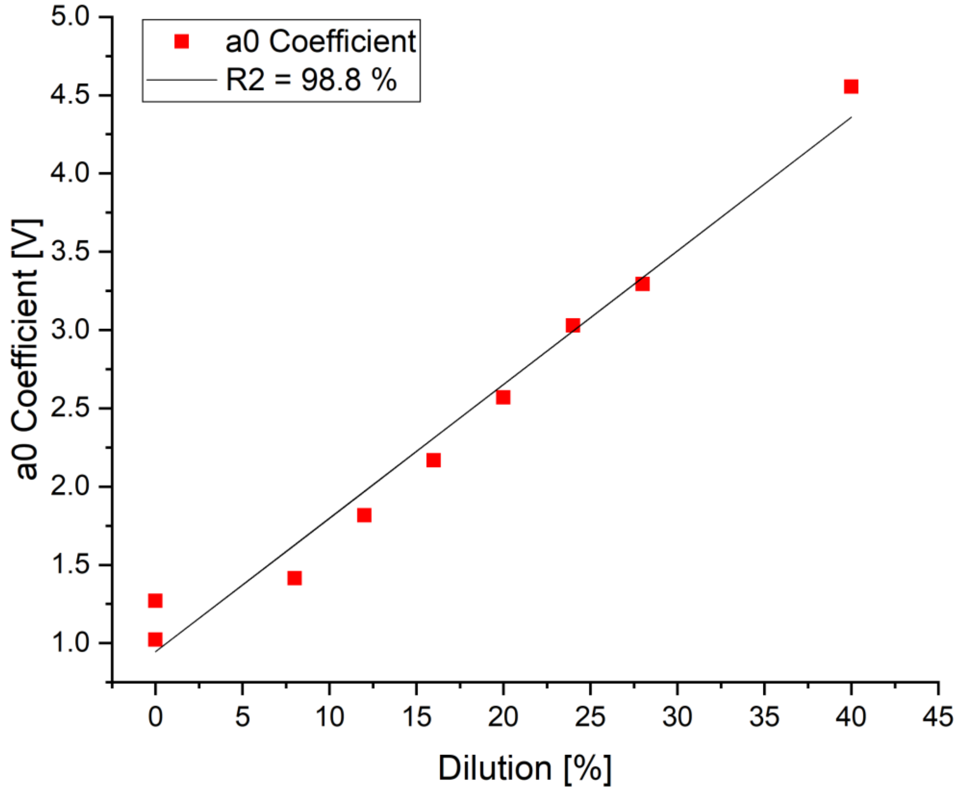

Figure S3. Calibration curve for hydrogen sulfide based on a serial dilution of a stock solution through which a small volume of gaseous hydrogen sulfide had been bubbled.

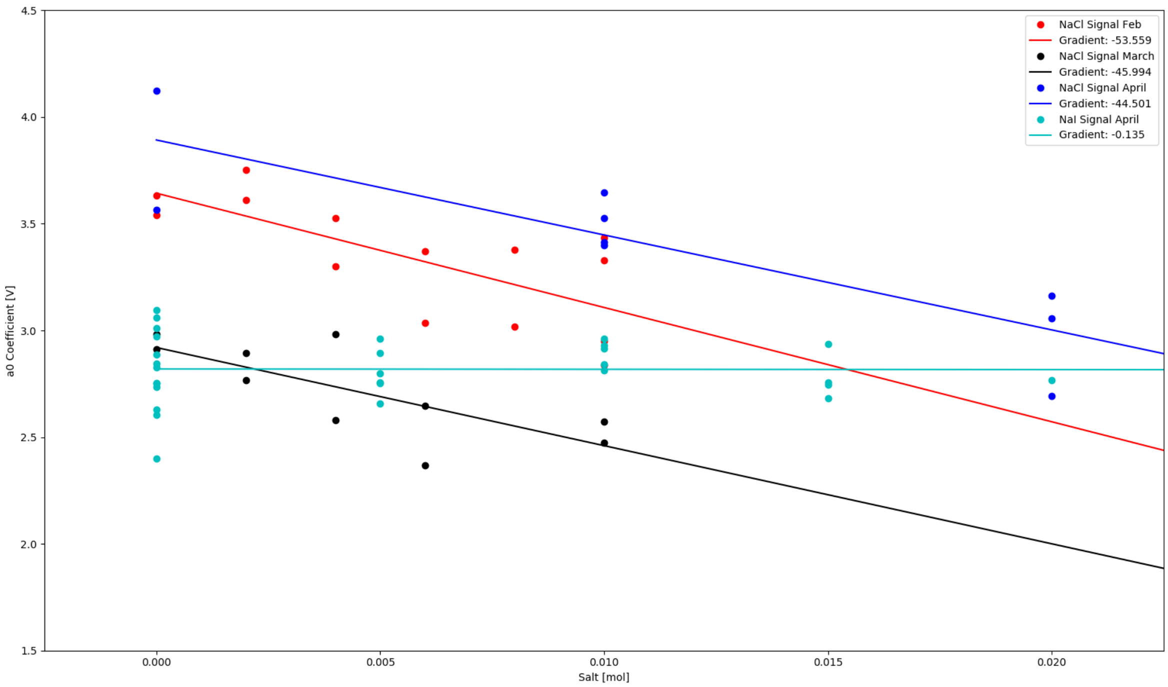

Figure S4. Influence of sodium chloride and iodide salt concentration on a_0 coefficients of 62 ppm ammonia.

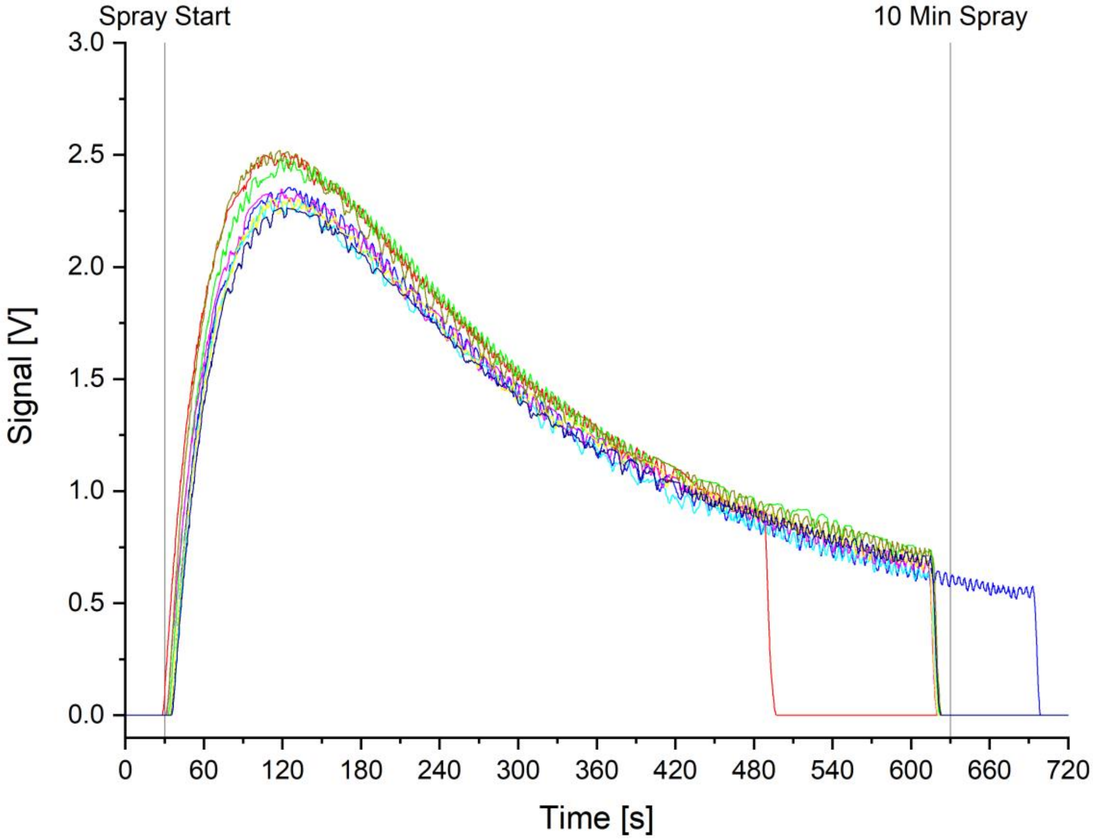

Figure S5. Signal from hydrogen sulfide sensor due to gas stream saturated with water. Synthetic air with 5% relative humidity measured until spray start at 30 s. Signal drop below 0.5 V shows the switch to purge gas stream at 5% relative humidity.

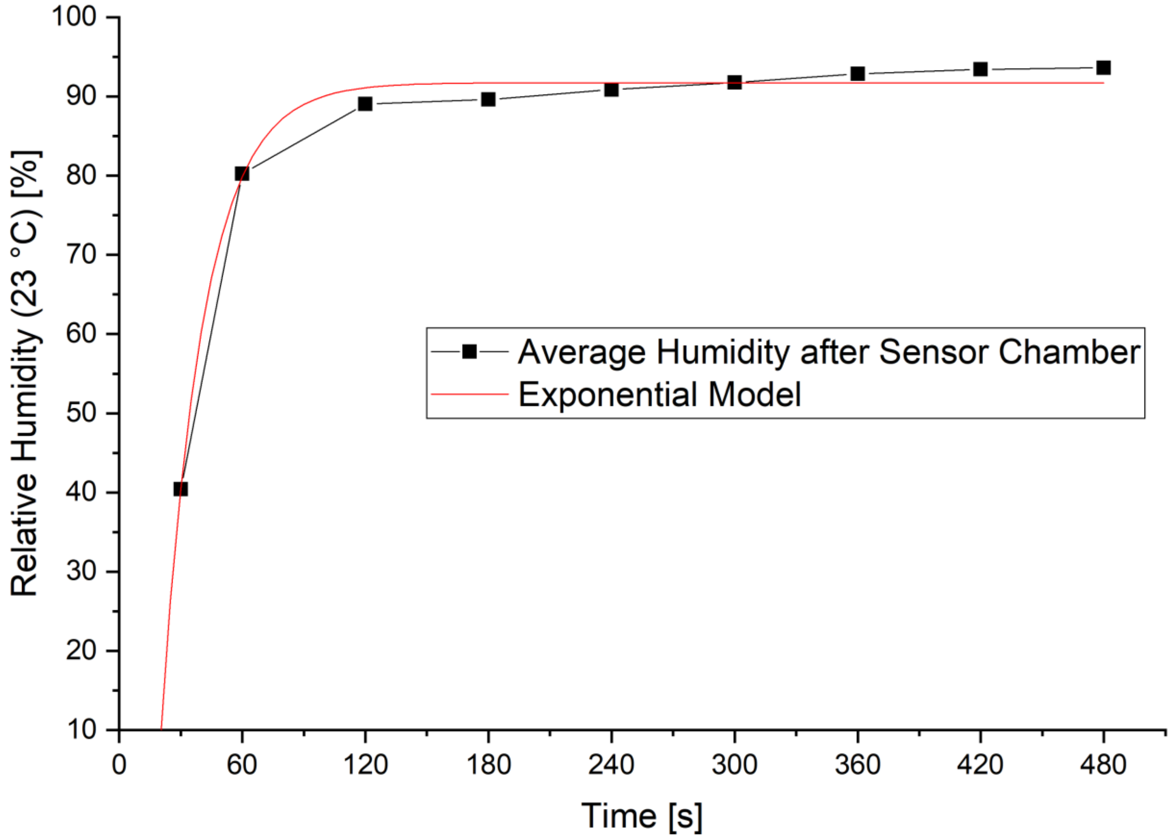

Figure S6. Change in humidity during spray measured by Testo 435 humidity probe. Exponential model rh = 91.73 * (1 − e^(−0.0488 * (t−18))) in the same form as Equation (5).
